# Beliefs and Attitudes of Hesitant Spaniards Towards COVID-19 Vaccines: “A Personal Decision”

**DOI:** 10.3390/healthcare13101199

**Published:** 2025-05-20

**Authors:** Andrea Langbecker, Daniel Catalan-Matamoros

**Affiliations:** Medialab Research Group, Department of Communication and Media Studies, Madrid University Carlos III, 28903 Getafe, Madrid, Spain; dacatala@hum.uc3m.es

**Keywords:** vaccine hesitancy, coronavirus, media, social networks, close people, antivax

## Abstract

**Background/Objectives:** Vaccine hesitancy has increased during the coronavirus pandemic, a period marked by the spread of disinformation and conspiracy theories about COVID-19 vaccines. This qualitative study aimed to explore the beliefs and attitudes of Spaniards towards COVID-19 vaccines and analyze the role of information sources in this process. **Methods**: Semi-structured interviews were conducted with 35 residents of Spain who exhibited varying levels of vaccine hesitancy. Through thematic content analysis, the narratives supporting vaccination-related decisions, as well as the influence and trust in information sources, were examined. **Results**: Reasons for getting vaccinated included perceptions of it being “almost an obligation” and “fear of illness and death”. Conversely, reasons for not getting vaccinated included “uncertainty about vaccines”, the belief that “the risk is not real”, and the perception that “vaccination is a personal decision”. Regarding vaccine-related information sources, interviewees expressed distrust of the media, particularly television, as they considered news about vaccine effectiveness and characteristics to be contradictory and constantly changing, which created uncertainty about its reliability. Most interviewees were unsure if social media influenced their decision not to get vaccinated. However, those who acknowledged its impact mentioned trusting sources such as people with a university education. Additionally, close contacts—particularly healthcare professionals—had a significant influence on the decision not to get vaccinated. **Conclusions**: This study shows that the decision not to vaccinate is shaped by personal beliefs and sources of information—particularly social media and close acquaintances, including healthcare professionals.

## 1. Introduction

Spain has been severely affected during the pandemic, ranking fifteenth worldwide as well as sixth in Europe in terms of the highest number of deaths from coronavirus in absolute terms. As of 20 October 2024, there have been 121,852 deaths recorded [1]. The country has 86% of the total population fully immunized as of June 2023. Despite this privileged situation, it faces challenges in achieving broader COVID-19 vaccine coverage, especially regarding booster doses. Significant differences are observed in certain age groups, particularly among the younger population. For example, only 51% of people aged 30 to 39 and 45% of those aged 20 to 29 have received the first booster dose, while the population aged 60 and above has a coverage rate of over 90%. Furthermore, these rates vary considerably across autonomous communities [2] which refer to the administrative divisions in Spain that have a degree of political and administrative autonomy.

These data may reflect the rejection or doubts among Spaniards when it comes to receiving the COVID-19 booster dose. According to a global survey conducted by [3] in 23 countries in 2022, 13% of Spanish participants showed hesitancy in getting vaccinated with the booster dose, ranking eighth among the surveyed countries with the highest vaccine hesitancy compared to the global study average of 12%.

Understanding why people have vaccine hesitancy is a complex task as not everyone behaves in the same way and their decisions are not always based on rationality. Some individuals may maintain a decision not to vaccinate over time and be inflexible about it, while others may initially resist getting vaccinated but later reconsider their decision. It is also possible for some people to vaccinate against certain diseases but refuse to do so with other vaccines [4,5].

Unlike other European countries that experienced declines in vaccine coverage in the years prior to the pandemic, Spain has a long history of population acceptance of immunization programmes evidenced by high rates of childhood vaccine coverage. Moreover, there is no organized and significant anti-vaccine movement in this country. However, during the pandemic, isolated cases of protests against COVID-19 vaccines have been reported, where some demonstrators took to the streets to protest against the Spanish government and the preventive measures implemented to combat the coronavirus, including vaccines [6]. Nevertheless, it should be noted that not all people who have vaccine hesitancy necessarily belong to anti-vaccine groups.

The anti-vaccine movement is not a new phenomenon, dating back to the 19th century, but it has gained strength with the use of social media which have become a sort of arena for the dissemination of misinformation, disinformation, alarmist content, and vaccine-related conspiracy theories [7,8,9].

This situation is exacerbated by the significant expansion of these platforms, especially during the pandemic, and the importance we give them in our daily lives. In Spain, for example, 85% of internet users between the ages of 12 and 74 use social media, representing nearly 30 million Spaniards [10].

This situation becomes even more complicated when false information goes viral and reaches a wide audience, making it difficult to debunk [11]. Anti-vaccine hoaxes and those that cast doubt on the safety of COVID-19 vaccines were frequent in Spanish tweets at the beginning of the pandemic [12], as well as fake news about vaccines and vaccination [11].

On the other hand, Spanish media outlets have played a particularly active role during the epidemic, generating a significant amount of informative content [13]. As the pandemic progressed, COVID-19 information gained more prominence and occupied an increasingly larger space on the covers of Spanish newspapers [14]. During the lockdown in 2020, there was an 8% increase in television consumption compared to 2019, reflecting the need for information during the pandemic in a period dominated by uncertainty [15].

In this regard, access to reliable sources of information plays a crucial role in combating infodemics and misinformation. People’s relationship with sources is marked by complexity and dynamism as the choice of one or multiple sources for information is closely linked to the level of trust people place in them. Individuals have consulted various sources to obtain information about the pandemic and vaccines [16], with social media usage standing out [17,18,19]. This reliance on social media has contributed to lower predisposition to vaccination compared to other sources such as healthcare professionals and official sources [7,20,21].

The importance of this study lies in the detailed analysis of the situation in Spain, a country that has experienced severe consequences in health, economic, and social terms due to the health crisis. In this context, vaccination has stood out as the most effective strategy to reduce the impact of the pandemic. Despite the progress made in immunization, Spain continues to face challenges in achieving sufficient vaccination coverage, especially concerning booster doses. Vaccine hesitancy, identified as a threat to public health worldwide [22], has raised concern among health authorities. Thus, understanding the role that information sources play in shaping vaccine-related decisions is crucial. This approach is essential to address the underlying causes of vaccine hesitancy and to promote greater public confidence in COVID-19 vaccination.

Previous surveys have been conducted with the Spanish population to analyze information consumption [23,24] and vaccine-related disinformation during the pandemic [25]. However, to the best of our knowledge, there are no qualitative studies that thoroughly investigate the relationship between vaccine hesitancy and the consumption of information about COVID-19 vaccines.

This type of study provides an opportunity to explore the nuances that influence vaccination decisions as well as to better understand patterns of information consumption and the role of media and social networks in that process. In general, previous research—mainly based on surveys—has focused on identifying which media people used and what kind of information they consumed, offering a broader but less detailed view of the phenomenon [23,24,25].

Moreover, although studies have been conducted in other countries, it is important to consider that people’s relationships with media and social media vary according to national context. Therefore, findings from other settings are not always directly applicable to Spain, which further reinforces the relevance and importance of the present investigation. This study aims to fill this gap by providing empirical qualitative evidence on the interplay between media consumption and vaccine hesitancy in Spain.

Based on the above, this study is grounded on the following research questions: (1) What role do sources of information play in the decision-making process about vaccine hesitancy among Spaniards? (2) What are the underlying beliefs behind vaccine hesitancy among Spaniards?

To address these questions, the aim of this study was to explore the beliefs and attitudes of Spaniards who exhibited some level of vaccine hesitancy regarding COVID-19 vaccines, as well as to analyze the role of information sources in this hesitancy.

## 2. Materials and Methods

### 2.1. Study Design

We conducted an exploratory qualitative study that allowed participants to express their beliefs, opinions, feelings, and ways of thinking and acting. In other words, it enabled them to reveal the interpretations they make about a specific aspect of their life experience [26], and in this study, specifically regarding COVID-19 and vaccine hesitancy.

We used semi-structured interviews based on a previously prepared script, which included both closed and open-ended questions. This approach allowed the interviewees to elaborate on their answers without being constrained by the wording of the questions. In some cases, follow-up questions such as “why” or “how” were used to delve into unforeseen issues that emerged during the interview [27].

### 2.2. Participants, Recruitment and Inclusion Criteria

A total of 35 participants (18 women and 17 men) were interviewed to ensure diversity in the sample. Interviewees were recruited through the AsuFieldwork company’s panel, specialized in qualitative research, as well as through social media. They were provided with a small compensation for their participation.

To participate in this study, interviewees had to meet the following requirements: (1) be over 18 years old; (2) reside in Spain; and (3) have some level of vaccine hesitancy towards COVID-19 vaccines. To ensure that all interviewees met this last criterion, three screening questions were asked. (1) Have you been vaccinated against the coronavirus? (2) Have you received the booster dose against the coronavirus? (3) When deciding whether to get vaccinated against COVID-19, did you have significant doubts about whether to get vaccinated or not?

Based on these screening questions, participants who fit the study’s target audience were selected: people who had not been vaccinated against COVID-19, people who had been fully or partially vaccinated but had not received booster doses and had no intention of doing so, and those who had been fully vaccinated and had received some booster doses but were unsure if they would continue with future booster doses. Candidates who did not exhibit any level of vaccine hesitancy towards the COVID-19 vaccine were excluded.

### 2.3. Study Overview and Researcher Contributions

This study is part of the PredCov project (multi-source and multi-method prediction to support COVID-19 policy decision-making), which is financed through an agreement between the Community of Madrid (Ministry of Education, Universities and Science) and Madrid University Carlos III for the direct grant of EUR 4,859,000 to fund the development of research activities on SARS-CoV-2 and COVID-19, funded with REACT-EU resources from the European Regional Development Fund, “A way of making Europe”. This paper has also received funding from the HEALTHCOM project coordinated by the University Carlos III of Madrid, and financed by the State Research Agency of the Ministry of Science and Innovation for the period 2023 - 2027, ref: PID2022-142755OB-I00.

The interview script was developed by the first author from Universidad Carlos III de Madrid and reviewed by four researchers from the Medialab Research Group at the same university. The project contracted the company AsuFieldwork to conduct the interviews, which were carried out under the guidance and supervision of the study’s authors. The data analysis, on the other hand, was conducted by the first author.

### 2.4. The Interview Guide

To verify if the script met the research objectives, four pilot interviews were conducted by the first author. These interviews helped assess the clarity and focus of the questions. Based on the feedback, adjustments were made to the script until the final version, which was approved by both authors. The pilot interviews were not included in the final sample.

The script was structured into four sections: (1) participants’ experience with COVID-19 vaccines; (2) media and COVID-19 vaccines; (3) social media and COVID-19 vaccines; and (4) other sources. In the first section, the questions aimed to explore the motivations of the interviewees for accepting or rejecting the vaccine. In the second section, inquiries were made about the consumption of information related to the topic and the level of trust and influence of the media in making vaccination decisions. The third section examined the use of social media as a source of information about vaccines and the interviewees’ behaviour regarding participation in debates about COVID-19 vaccines on social media. Additionally, it was investigated whether social media influenced vaccine hesitancy. The fourth section explored the use of other information sources and their influence in making decisions related to COVID-19 vaccines.

### 2.5. Interviews

Telephone interviews were conducted during the months of October, November, and December 2022, which allowed the interviewing of individuals from 14 autonomous communities in Spain. The company AsuFieldwork was responsible for conducting the interviews, under the guidance and supervision of the researchers of this study. Each interview had an average duration of 40 min and was recorded for subsequent analysis. The interviews were also transcribed, which were reviewed by the first author of the study after listening to the audio recordings.

### 2.6. Ethics

Interviewees were informed about the nature of the study, its objectives, and were assured that they had the right to refuse to answer questions or interrupt their participation at any time during the interview without needing to justify their decision.

All participants provided their consent to participate in the research, and their data were fully anonymized. To meet ethical requirements, the study was approved by the Ethics Committee of (anonymous) under the CEI_22 protocol.

To ensure the right to information, participants were provided with a text detailing the benefits of vaccination and booster doses, along with links to access more information on the topic at the end of the interview.

### 2.7. Data Analysis

For the data analysis, a content analysis technique was employed—which involves identifying the core meanings present in the communication and determining their relevance to the established analytical objectives [28]. All interviews were carefully read and reread to identify similarities, differences, and specific aspects present in the texts. Relevant content was highlighted and quotes were extracted from the interviews and grouped into nodes. This process was conducted manually using NVivo 11 software, a tool that aids in organizing and systematizing data quality studies [29]. In addition, diagrams were generated using this programme. The analysis aimed to delve deeper into understanding the underlying meanings of the narratives constructed to support vaccination-related decisions. Here, narratives are understood as: “a fundamentally human way of giving meaning to experience. In both telling and interpreting experiences, narrative mediates between an inner world of thought–feeling and an outer world of observable actions and states of affairs” [30].

## 3. Results

The sample consisted of 18 women and 17 men, with an average age of 40 years, and educational levels of university degree (65%) and secondary school diploma (34%). Regarding their religious beliefs, 51% consider themselves Catholic (practicing and non-practicing), 22% are atheists, 20% are agnostic, and 5% do not identify with any of the aforementioned options. The majority of the interviewees identify themselves as centre, centre-left, and centre-right (Table 1).

There were 23 participants who had been vaccinated (with a full schedule; full schedule and one booster dose; full schedule and two booster doses; partial schedule), and 12 people had not been vaccinated.

All participants showed different levels of vaccine hesitancy depending on the stage they were at. Among the 23 vaccinated interviewees, only 8 had received the first booster dose, while the rest had not and had no intention of doing so, as highlighted in the interviewee I1’s statement: “I understand that in the first doses there wasn’t the complete, total information, but now it’s the fourth dose, come on! Why? Give me reasons, explain it to me”.

However, out of the eight who have received the first booster dose, three have already decided not to get the second booster dose, and five have doubts about whether they will do it.

Those who have not been vaccinated can be grouped into two main categories: (1) Those who have specific doubts related to the COVID-19 vaccine; some interviewees highlighted that they had been vaccinated throughout their lives but resisted doing so with the coronavirus vaccine. (2) To a lesser extent, there are participants who are against vaccines in general.

Of the 12 unvaccinated interviewees, 10 are still considering not getting the vaccine: “No, no, no! I won’t get it, neither me nor my high-risk daughters, nor my grandmother, nor my husband, nor anyone” (I9). Two of the interviewees expressed the possibility of changing their minds in the future, as is the case with participant I8. “[Wait] a few years to see how the vaccines are being consolidated and what side effects they have. Wait for the side effects”.

Vaccination decisions regarding COVID-19 are influenced by narratives that support them. We have identified 4 narratives in favour and 11 narratives supporting the decision not to get vaccinated, as illustrated in Table 2.

### 3.1. Narratives That Support Decisions to Get Vaccinated

#### Narratives “Almost an Obligation”, “Fear of Illness and Death” and Protecting Society

The narrative “almost an obligation” includes testimonies from people who initially had little interest in getting vaccinated but felt pressured to do so due to social influence and to “follow the crowd” (I3). Moreover, the work environment and imposed restrictions also played a significant role in decision-making as people felt compelled to get vaccinated if they wanted to return to normal and access activities such as going to restaurants and travelling. According to interviewee 33, there is strong social pressure to conform to what everyone else is doing. Those who do not comply may be perceived as irrational or out of place. For this reason, the interviewee felt compelled to get vaccinated and follow the majority: “It annoys me because, well, you feel like a kind of sheep, but what can you do?” (I33).

In the narrative “fear of illness and death”, some participants described how uncertainty and limited knowledge about the severity of COVID-19 influenced their decision to get vaccinated. For example, interviewee 26 explained that their choice was primarily driven by fear—both of the disease itself and of its then-unknown nature and apparent virulence during the early stages of the pandemic.

However, it is highlighted that few accounts linked the decision to get vaccinated with the prevention and care of the collective, although there are some exceptions. For example, interviewee I34 expressed a belief in vaccination at the beginning of the pandemic to end the health crisis and how research in this field could help us overcome diseases: “I believed that, like all of Spain, we had to contribute our grain of sand” (I34).

### 3.2. Narratives That Support Decisions to Not Get Vaccinated

#### 3.2.1. Narrative “Uncertainty Towards Vaccines”

The motivations behind the narratives against vaccination are varied as the interviewees used more than one argument to support this decision. In the narrative “Uncertainty towards vaccines”, the predominant perception was the lack of information about the vaccines, their components, and specifically about the possible effects on the body, the fear of side effects, and the possibility of death. The fear of the unknown, of not knowing what they will face if they get vaccinated, has been an important factor in the decision not to get vaccinated. As interviewee 32 expressed, they felt relieved not to have been vaccinated with something whose long-term effects on the body are still uncertain, mentioning concern about what might happen in a few years and citing the increasing number of unusual cases of cancer and strokes which they found troubling.

In the same vein, some interviewees expressed their lack of trust in vaccines due to their rapid development in a short period of time, compared to previous vaccines that took longer to develop. They considered these vaccines to be experimental and did not want to be used as “guinea pigs”. The interviewees argued that, for these reasons, it was impossible to know what the potential health consequences might be, making the vaccines unreliable.

Furthermore, some interviewees did not accept the fact that a vaccine against the coronavirus had been developed in such a short period of time, especially compared to other diseases for which a vaccine has not yet been found, such as cancer. All of this caused insecurity regarding the COVID-19 vaccines.

#### 3.2.2. Narrative “The Risk Is Not Real”

In the narrative “the risk is not real,” some interviewees did not get vaccinated, either with the full schedule or with booster doses, because (1) they did not perceive the virus as a threat to their health; (2) others argued that the coronavirus was “similar to the flu” and therefore did not see the need to get vaccinated; (3) some people who do not have pre-existing health issues believe they are not at risk of suffering complications from the disease; (4) lastly, there are those who chose to deny the existence of the pandemic. On one hand, by not informing themselves about the topic, they considered that the problem simply did not exist: “The vaccine and COVID issue was not talked about in my house; as there was no television, there was no fear, there was no panic, just being obsessed with that topic (I6)”. On the other hand, there was the perception that the virus was not as aggressive and deadly as it had been reported.

#### 3.2.3. Narrative “Vaccination Is a Personal Decision”

The interviewees also took the decision to get vaccinated for themselves and believed that vaccination should not be an imposition from the government and society, as perceived in the narrative “Vaccination is a personal decision”. In this narrative, the opinion is reflected that each person should have the right to decide whether to get vaccinated or not, without external imposition. The interviewees expressed that vaccination is a personal matter and that they should have the freedom to make that decision based on their own beliefs and circumstances:

That’s where I have to choose whether I lose my trip and lose my freedom for a vaccine, that makes me angry, that generates more than anything else, dissatisfaction, it makes me angry to be subjected to that [...]. I should decide about my body, just like one decides about abortion or whatever (I2).

#### 3.2.4. Narrative “Hidden Interests”

The narrative “hidden interests” reflects conspiratorial ideas in which it is argued that decisions to produce vaccines have been made with the aim of benefiting pharmaceutical companies and certain individuals economically. These facts have generated reluctance towards vaccines among some interviewees. For instance, interviewee 20 recounted discovering that a high-ranking authority in the European Union, Ursula von der Leyen, has a husband who is or was a senior executive at a vaccine manufacturing company. They perceived it as suspicious that the same vaccine produced by that company was, in their words, “practically forced upon us,” which they saw as more than just a coincidence.

It is important to highlight that, in general, each person built multiple narratives (both in favour and against) that gave meaning to their decisions.

### 3.3. Sources of Information and COVID-19 Vaccines

#### 3.3.1. Media

A total of 71% of the interviewees have utilized various types of media, mainly newspapers and television, to gather information about vaccines. However, they have also resorted to searching for information on the internet and through social media. In the case of internet search engines, it is observed that the interviewees looked for content about vaccines without paying much attention to the source of the information, but rather gave more importance to the content itself. It is interesting to note that the majority of the interviewees were not loyal to specific media outlets and have lost trust in the media over time. As interviewee 9 explained, their trust in the media has declined significantly: at first, they believed what was being reported, but now they feel they can no longer trust anything the media says.

The interviewees expressed their mistrust towards the media because they considered that the focus of the news regarding the effectiveness and characteristics of the vaccine was contradictory and varied over time. This situation generated insecurity about the truthfulness of the news as they did not know if the information would completely change the following week.

Furthermore, the interviewees highlighted the excess of information as a factor that contributed to generating doubts and causing anxiety. They also mentioned the politicization of the media and sensationalism in the pursuit of audiences as factors that influenced the lack of trust. Furthermore, the interviewees expressed a higher degree of rejection towards television compared to other media outlets: “I haven’t watched television for 3 years, so the media is the terror of society” (I6).

This mistrust towards television originates from the perception that this media manipulates or omits information, generates fear in people, and presents news that is inconsistent with the reality of vaccines: “I have had people from within, I have seen pregnant women die, I have seen children die, and they say on television that pregnant women, children, and nothing die. All lies” (I11). Several interviewees perceive television as a media outlet that is more subordinate to the government and, therefore, distrust the truthfulness of journalistic coverage on the subject. However, the interviewees expressed more trust in news when it came from the press.

For some participants, the media coverage on vaccines was a “monotheme”, without expanding the information in a balanced manner. According to this perception, everything that was heard was in favour of vaccination, as if it were an obligation imposed by law and supported by the media on behalf of politicians and public organizations. This uniformity in the message generated mistrust in the possible hidden interests behind this discourse.

Some participants also expressed the opinion that the media carried out a kind of witch hunt against those who had not been vaccinated, which fuelled hatred towards them, even though they had followed other preventive measures. For example, interviewee 29 described how, despite wearing a mask and avoiding social outings, they still felt unfairly judged solely for not being vaccinated.

Another relevant aspect to highlight is the shift in media coverage on vaccines. Despite an initial widespread dissemination promoting vaccination, some interviewees have noticed a decrease in media attention on the topic, even though cases continue to occur (I30).

#### 3.3.2. Social Media

The use of social media to gather information about vaccines was a common practice among all interviewees. The majority of them are users of multiple social media platforms. It was also observed that individuals in the age range of 40 to 49 years old were the ones who used multiple social media platforms to seek information about vaccines.

**Figure 1:**
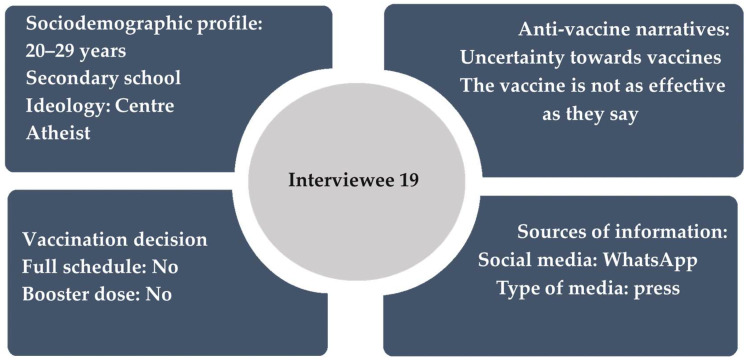
Sociodemographic profile, vaccination decision, anti-vaccine narratives, and use of information sources of I9.

The information of interest to several participants was mainly the adverse effects of the COVID-19 vaccines and concerns about potential illnesses, male sterilization, or female infertility. Some of them have shared this type of content, like the case of I9, who used TikTok as a source of information on the topic. Whenever they came across a video related to the side effects of vaccines, they would share it: “The stories of people... there’s a 15-year-old girl who had to have both legs amputated because she had kidney failure from the vaccines” (I9). Figure 1 summarizes the sociodemographic profile, vaccination decisions, and information consumption behaviour of this interviewee.

The participant I21 found accounts of people claiming to have suffered adverse effects because of the vaccine. Based on these experiences, she began to identify similar symptoms in herself, but long after having received the full schedule. However, the interviewee has not received medical confirmation as she did not want to discuss it with her primary care doctor for fear of being considered a bit “paranoid”. Nevertheless, after believing she experienced side effects, she decided not to receive any booster doses.

Some users sought content on social media to contrast what was being said in conventional media, thus seeking another interpretation of the facts:

It’s like the movie starring Leonardo Di Caprio, Don’t Look Up, where a group of scientists warns the government that a meteorite is going to fall, and they take it as a joke. Well, here it’s the same, but what happens is that in this case, the meteorite is false, and the decisions made about it could be different (I20).

In the same line, interviewee I4 mentioned having seen a video where a person claimed to have become magnetically attracted to metal objects after receiving the vaccine, as alleged evidence that a microchip had been injected into them to control people. Although he considered this claim to be nonsense and a hoax, he decided to try it himself. However, when he put a spoon on his arm and noticed it sticking, it gave him an “unsettling feeling”.

In the case of interviewee 20, social media—particularly Twitter (now X)—is perceived as a space where it is possible to follow individuals who possess knowledge but are not aligned with mainstream narratives. He sees the platform as a refuge for minority voices—those who are often silenced or excluded from public debate but who, in his view, have valuable insights to offer. These individuals, he believes, turn to less conventional and less popular platforms like Twitter as an alternative space for expression and information sharing.

Most of the interviewees are not clear if social media has influenced their decision not to get vaccinated. However, those who have acknowledged the influence of social media highlighted the role of some sources they consider reliable, such as those with a university level of education. For example, biologist and science communicator Fernando López Mirones was mentioned, who has 32 thousand followers on Facebook, and pharmacist María García Alonso, with 88.5 thousand followers on Instagram. López Mirones has shared videos questioning mRNA vaccines and has participated in programmes to discuss the topic. On the other hand, García Alonso has shared various topics related to vaccines, but has mainly focused on the role of natural immunity against the virus and the importance of vitamin D, sharing studies, news, and other posts on these subjects.

Some interviewees have expressed criticism towards the denialist content and misinformation present on social media, as they consider them not to be reasonable arguments. These interviewees recognize that the virus is real and that there is scientific evidence of its existence. However, they have chosen not to get vaccinated because they believe that vaccination should not be something mass imposed but should take into account each individual case.

Most of the interviewees have chosen not to share vaccine-related content on social media, as they consider the decision not to get vaccinated to be a personal one and do not wish to influence others’ decisions. Furthermore, some participants avoided engaging in debates on the topic due to the perception that expressing anti-vaccine opinions is socially frowned upon.

#### 3.3.3. People in Their Social Circle and Healthcare Professionals

Several interviewees have also admitted the influence of family members and friends on their decision not to get vaccinated, whether through receiving shared content or based on accounts of experiences with vaccines from these individuals. Additionally, the importance given to these sources increased if these close people were healthcare professionals.

Some of them had received the vaccine but expressed that they “would not get it again if they could go back in time” (I14), while others had not received it, as mentioned by interviewee 21 about her friend: “Well, being a doctor, she hadn’t gotten it”.

Few interviewees have mentioned healthcare professionals (outside of their personal circle) as a source of information that has influenced their decisions about vaccines. Interviewee E19 expressed his opinion that doctors themselves did not agree on vaccines. According to him, there was no consensus among doctors, as some had recommended getting vaccinated while others advised against it. This lack of agreement led him to the conclusion that no one really knows what to do about vaccines.

Some interviewees also expressed a lack of trust in the statements of healthcare professionals, such as the case of Fernando Simón, a Spanish epidemiologist and spokesperson for the Ministry of Health during the coronavirus crisis. According to interviewee 33, it was difficult to believe in the credibility of someone who would say one thing one day and another thing the next, especially when it came to a spokesperson of a country regarding such a serious issue. “Simón’s statements were grotesque, ridiculous, and a mockery” (I33). These inconsistencies in Simón’s statements have also raised doubts about trust in COVID-19 vaccines.

Moreover, it was observed that some interviewees highlighted their university education as support for not getting vaccinated: “First, because I have a PhD in Biology and I know how a vaccine is developed […]”. Although it may seem contradictory, they also sought information about vaccines in scientific journals to justify their decision not to get vaccinated. “I went to The Lancet’s website, where scientific articles are published. I informed myself, read a few, formed an opinion, and that was it” (I35).

## 4. Discussion

This study conducted 34 semi-structured interviews with Spanish residents who exhibited some level of vaccine hesitancy towards COVID-19, with the aim of exploring the beliefs and motivations underlying these doubts. To achieve this, the narratives supporting these decisions, whether in favour or against vaccination, were investigated, as well as the role played by sources of information in shaping these narratives.

The adverse effects of vaccines are extremely rare, and the vast majority of people only experience mild and temporary side effects. For example, the AstraZeneca and Janssen vaccines against SARS-CoV-2 can cause autoimmune thrombosis in association with thrombocytopenia. The frequency of this adverse effect is very low, at 0.000065%, according to data from the European Medicines Agency. On the other hand, the risk of death from COVID-19 is higher, estimated at 0.4% for individuals under the age of 55 [31].

Despite these promising data, the narrative of “Uncertainty towards vaccines”, which encompasses the fear of adverse effects, was the most prominent in relation to the decision of not getting vaccinated or not receiving the booster dose. These findings confirm a trend identified in the Spanish population before the start of COVID-19 vaccination: lack of efficacy and safety as well as potential adverse effects were identified as reasons for not getting vaccinated [32]. García-Iglesias et al. [33] highlight that during a health crisis, people tend to focus on threatening information, which, along with a reduced perception of control, can increase the likelihood of experiencing symptoms such as anxiety, depression, and emotional exhaustion. This effect is amplified in situations of loneliness and isolation.

This narrative is linked to another one, which is “the risk is not real”. Here, it is about how people perceive and respond to risk, which is subjective and influenced by fear, beliefs, and previous experiences. However, this perception does not correspond to the actual risk of COVID-19 vaccines, which is quite low. For the interviewees, there was more risk in getting vaccinated than in getting sick. This perception of risk affects trust in science, authorities, and the media [8].

Understanding where the influence behind the decision not to get vaccinated comes from is complex, as this choice is usually shaped by multiple factors. Most interviewees were unable to clearly identify which sources played a more significant role in this process. However, there are interviewees who did point to social media as one of the main influences. These findings are consistent with those of Sallam et al. [16] who identified a link between uncertainty about COVID-19 vaccines and the use of social media as the primary source of information on the topic.

During the pandemic, there was a considerable increase in content related to COVID-19 vaccines, beginning with the early stages of vaccine development and intensifying during the vaccination campaigns in Europe. This surge in information led to a parallel rise in disinformation, which became a persistent issue [34].

This type of content is particularly harmful, as it can influence individuals who are already hesitant about vaccines, potentially leading them to reject vaccination altogether [35]. In line with this, Hu et al. [36] argue that social media platforms with large user bases have disrupted traditional channels of vaccine communication, allowing anti-vaccine individuals to disseminate misleading messages to targeted audiences whose views on vaccination may be more susceptible to change. Anti-vaccine groups are noted for their strong internal cohesion and high levels of activity, even though their capacity to spread content virally is relatively limited [37]. Similarly, Reno et al. [20] found that the use of social media increased vaccine hesitancy, whereas information from official sources had the opposite effect.

Our findings also show that content related to adverse effects played a significant role in shaping public perceptions. According to Romate et al. [38] the main psychological factors contributing to COVID-19 vaccine hesitancy included concerns about safety and side effects, trust in the vaccine itself, in government authorities, and in healthcare professionals, as well as scepticism about the vaccine development process and belief in conspiracy theories.

Other research further supports these observations. Park et al. [7], for instance, found that individuals who relied on social media for information tended to perceive lower risk associated with COVID-19 and were less inclined to get vaccinated, compared to those who trusted healthcare professionals.

The results of this study also highlight the role of conspiracy-related ideas in promoting vaccine hesitancy. These findings align with those of Kant et al. [39] who reported that anti-vaccine groups and individuals deliberately spread conspiracy theories—such as the claim that vaccines could damage DNA or permanently alter human genes—in order to foster distrust. They also observed that much of the misinformation found on Facebook originated from international sources and contributed to widely circulated global conspiracy narratives about COVID-19 vaccines. These findings reinforce the growing body of evidence indicating that belief in conspiracy theories is a strong predictor of vaccine reluctance. In this same context, the 26 min documentary *Plandemic*, released in May 2020, went viral by promoting such misinformation—including the dangerous belief that COVID-19 vaccines are harmful. The video’s creator attracted 130,000 followers within just one month of its release [40].

Our results indicate that, in the case of influencers, the positions taken by individuals with university-level knowledge about vaccines have contributed to vaccine hesitancy. The perception of the interviewees is that these influencers understand how vaccines work. It is important to note that they are not scientists involved in vaccine research projects. However, the interviewees perceive them as experts in the field. Their influence lies in presenting a thought that is perceived as opposing the dominant scientific thinking or, according to the interviewees, amplifying existing knowledge on the subject and therefore generating trust. It is important to note that a well-coordinated network involving health communicators from scientific centres and health institutions, along with informed influencers on social media and fact-checking sources could more effectively prevent the spread of misinformation about vaccines among the public [41].

Furthermore, in the case of interviewees who did not acknowledge the influence of social media on their decision-making, they still exhibited behaviours that support this idea. For example, the belief that vaccines contain microchips or have some purpose of controlling people. Although this conspiracy theory has been discredited and lacks evidence, it generated doubts in some of the interviewees. The same occurred with those who identified with narratives from individuals claiming to experience adverse effects due to vaccines and subsequently started experiencing these symptoms themselves.

In all these cases, the role of disinformation in increasing vaccine hesitancy is identified. According to Larrondo-Ureta et al. [37], anti-vaccine discourse is spread through alternative media and shared content on social media, confirming that having access to quality information is one of the main measures against disinformation.

Close acquaintances were also an important source of information and were considered major influences. Among these, family members and friends—particularly those working in the healthcare field—stood out. It is expected that they would support vaccination, and if they do not, it is interpreted as a sign that the vaccine poses a real health risk.

The willingness of healthcare professionals to recommend vaccine use may be influenced by their experience with infectious diseases, their consequences, and their sequelae.

A study conducted with American physicians at various stages since their graduation from medical school revealed a 15% decrease in confidence in vaccine safety for every five-year interval since that period. This suggests that the perception of risks and benefits of immunization differs between newly trained doctors and their older colleagues, which certainly impacts how they recommend vaccines to their patients [42].

David et al. [43], for example, compared the value placed on close sources to that of healthcare professionals. In a qualitative study conducted with anti-vaccine individuals in Romania, the authors observed that these individuals trusted others more than doctors regarding vaccines, highlighting the importance of individualized information and documentation on the subject.

Similarly, De Araújo et al. [44] emphasizes the importance of implementing strategies to increase confidence in vaccination among healthcare workers, while considering differences in occupations and work environments. The authors suggest that improving vaccination-related content in training and continuing education activities and facilitating access to vaccinations in the workplace are crucial elements for reducing vaccine hesitancy among healthcare workers.

### 4.1. The Rejection of the Traditional Media

The media plays a crucial role in disseminating information to society and has been very active during the pandemic. More than 85% of Spaniards closely followed news about the pandemic and considered the media as one of the most important sources to obtain information on the topic [24]. In the same line, Mosteiro-Miguéns et al. [23] found that the internet (73%) and television (49.8%) were the most used sources by Spaniards to seek information on the subject. However, in the case of this study with vaccine-hesitant people, the majority showed a great distrust towards the media, both for those who used them and those who did not. According to Casero-Ripollés et al. [25], the distrust towards the media and politicians during the pandemic is a result of disinformation.

In our study, this distrust originates from the perception of inconsistency and unreliability in the information provided, especially through television. According to the interviewees, the fact that the media prioritizes a pro-vaccine approach generates mistrust, as journalistic coverage is expected to present diverse perspectives on a given topic, a historical norm related to source balance. However, the use of this norm can favour the dissemination of misinformation related to scientific topics [45]. In the case of media coverage on vaccines and autism, for example, the media often present arguments both in favour and against [46]. Nevertheless, Dixon and Clarke [46] argue that by presenting contradictory opinions in a balanced manner regarding the vaccine–autism controversy, there is a risk that readers may reach erroneous conclusions about expert knowledge regarding vaccine safety, which could have a negative impact on their vaccination intentions.

Although COVID-19 vaccination is not mandatory in Spain, the interviewees did not perceive it that way. They saw it as an external imposition and defended individual autonomy and the right to make decisions about their own bodies. Despite the importance of respecting autonomy and individual freedom to decide about one’s health, decisions related to public health, such as vaccination, have an impact beyond the personal sphere. Mass vaccination is an effective strategy to prevent diseases and protect the community as a whole.

### 4.2. Limitations

This qualitative study has some limitations that are important to consider. Although conducting interviews by phone makes it possible to reach more people, it somewhat limits the ability to establish a connection between the interviewer and the interviewee and there may be a loss of information. It is also important to note that it is impossible to guarantee that all participants were completely honest in their responses. However, unlike surveys, interviews provide participants with greater freedom to express their thoughts in depth. The interviewers were instructed to create a trusting environment and reassure participants about this. Furthermore, the sample was intentionally composed of individuals who had expressed some degree of vaccine hesitancy from the beginning. This means that the participants had already acknowledged their hesitancy, and this information was not perceived as particularly sensitive during the interviews.

Although the study focused on individuals with university education and in the middle-aged group, it is important to note that the goal of qualitative research is to delve into the analysis of specific groups, which differs from what is addressed in quantitative research. In other words, this study explored the decisions made by people with this particular profile. Additionally, our findings should not be extrapolated to other contexts, as each culture has its own specificities regarding vaccine hesitancy and the role of information sources in this topic.

These limitations should be taken into account when interpreting the findings, and it is suggested that future studies investigate the topic in other countries in order to obtain a more comprehensive and representative picture of the different perspectives and experiences related to vaccine hesitancy and the influence of sources on vaccination decision-making.

### 4.3. Practical Implications

The results of this study point to the need to promote health literacy, which involves the knowledge, motivation, and skills necessary to seek, understand, evaluate, and apply health-related information [47]. Low health literacy is a public health problem that has been exacerbated during the coronavirus pandemic [48]. A study that analyzed the association between health literacy, knowledge about COVID-19, and information sources, for example, identified that higher health literacy was associated with greater knowledge about the disease and the use of more sources to obtain information [49]. The authors also emphasize the importance of improving health literacy and providing accurate knowledge about COVID-19 in order to prevent being influenced by disinformation.

For this reason, it is necessary for the media to make an effort to incorporate an educational perspective into health-related information, especially in news and features. This will allow for the generation of quality scientific dissemination, promote greater prevention, and contribute to a stronger education of citizens [50]. Furthermore, it is essential for the media to be transparent in their coverage and provide information based on solid scientific evidence to promote trust and public understanding of vaccines. Another relevant aspect is the continuity of coverage on the topic, including updates on the vaccination campaign, the benefits of vaccination, and the importance of booster doses. The decision not to receive a booster dose can have implications for personal and community protection against COVID-19. With this strategy, it would prevent the topic from being forgotten and ensure that the population is informed and able to make informed decisions regarding COVID-19 vaccination.

Health organizations must also improve communication with the population by using media that can easily reach people, such as social media, utilizing well-known individuals, influencers, etc. It is important to promote a broader understanding of the importance of vaccination, both in terms of individual and collective benefits, to encourage greater acceptance and participation in vaccination programmes. Communication must be fluid and transparent. This is crucial so that when an event of a similar magnitude to the COVID-19 pandemic occurs, a relationship of trust has already been established. On the other hand, it is important to consider communication campaigns that are closer to the population and targeted to specific audiences.

## 5. Conclusions

The study identified various motivations behind vaccine hesitancy towards COVID-19. While some people get vaccinated out of obligation or for protection, others do not due to uncertainty about adverse effects, distrust in information sources, and risk perception. Disinformation, especially on social media, has played a key role, fueling conspiracy theories that generate doubts about the vaccine. Social media and stories from close acquaintances, including healthcare professionals, have a significant influence on vaccination decisions, highlighting the importance of promoting accurate information. Many interviewees expressed distrust towards traditional media, emphasizing the need to improve transparency and objectivity in health-related coverage. This study reveals that decisions not to vaccinate are influenced by beliefs, past experiences, and information sources, making it crucial to promote health literacy to equip individuals with tools to make more informed vaccine decisions.

## Figures and Tables

**Table 1:** Sample Characteristics.

Interviewees	Age	Education Level	Ideological Thinking	Religious Thinking
I1	45	Secondary School	Centre	Atheist
I2	39	Higher Education	Centre-left	Agnostic
I3	24	Secondary School	Left	Atheist
I4	45	Higher Education	Centre-right	Catholic
I5	67	Secondary School	Other	Catholic
I6	49	Higher Education	Centre	Other
I7	25	Higher Education	Centre-right	Catholic
I8	53	Secondary School	Right	Catholic
I9	28	Secondary School	Centre	Atheist
I10	20	Higher Education	Centre-right	Atheist
I11	58	Secondary School	Other	Catholic
I12	52	Secondary School	Centre-left	Atheist
I13	28	Higher Education	Centre-left	Atheist
I14	61	Higher Education	Centre-left	Catholic
I15	20	Higher Education	Centre	Agnostic
I16	32	Higher Education	Centre-left	Catholic
I17	51	Higher Education	Centre-left	Agnostic
I18	51	Secondary School	Right	Catholic
I19	36	Higher Education	Left	Catholic
I20	47	Higher Education	Centre-left	Agnostic
I21	55	Higher Education	Centre-right	Catholic
I22	21	Higher Education	Centre-left	Agnostic
I23	49	Higher Education	Centre-right	Other
I24	40	Secondary School	Centre	Catholic
I25	40	Higher Education	Right	Catholic
I26	46	Secondary School	Centre	Catholic
I27	29	Higher Education	Left	Agnostic
I28	38	Secondary School	Centre	Atheist
I29	32	Higher Education	Centre-right	Agnostic
I30	33	Secondary School	Right	Catholic
I31	43	Higher Education	Centre	Catholic
I32	49	Higher Education	Centre	Catholic
I33	60	Higher Education	Right	Atheist
I34	46	Higher Education	Other	Agnostic
I35	19	Higher Education	Far right	Catholic

**Table 2:** Overview of the narratives supporting interviewees’ decisions regarding COVID-19 vaccines.

	Narratives That Support Decisions to Get Vaccinated	Narratives Supporting the Decision Not to Get Vaccinated
Types of narratives	- Fear of illness and death - Almost an obligation - Protecting the family and friends - Protecting society	- Uncertainty towards vaccines - The risk is not real - Vaccination is a personal decision - Hidden interests - Other measures work better than vaccines - No one influences my opinion - Uncertainty towards the virus - Lack of convincing information - “I don’t get vaccinated, but I’m not a denier” - The vaccine is not as effective as they say - I already have immunity

## Data Availability

Data are unavailable due to privacy and ethical restrictions.
